# Microarray-Based Avidity Assay for Assessment of Thyroid Autoantibodies

**DOI:** 10.3390/diagnostics15030341

**Published:** 2025-01-31

**Authors:** Elena Savvateeva, Vera Sokolova, Marina Yukina, Nurana Nuralieva, Elena Kulagina, Maxim Donnikov, Lyudmila Kovalenko, Maria Kazakova, Ekaterina Troshina, Dmitry Gryadunov

**Affiliations:** 1 Center for Precision Genome Editing and Genetic Technologies for Biomedicine, Engelhardt Institute of Molecular Biology (EIMB), Russian Academy of Sciences, 119991 Moscow, Russia; len.savv@gmail.com (E.S.); sokolovavera99@mail.ru (V.S.); elenka176@yandex.ru (E.K.); 2 Endocrinology Research Centre, Ministry of Health of Russia, 117292 Moscow, Russia; kuronova@yandex.ru (M.Y.); nnurana@yandex.ru (N.N.); impdoctorx@gmail.com (M.K.); troshina@inbox.ru (E.T.); 3 Department of Children’s Diseases, Medical Institute of Surgut State University, 628400 Surgut, Russia; donnikov@gmail.com (M.D.); kovalenko_lv@surgu.ru (L.K.)

**Keywords:** autoimmune thyroid diseases, multiplex immunoassay, protein microarray, thyroid autoantibodies, avidity

## Abstract

**Background/Objectives:** The aim of this study was to evaluate the avidity of thyroid autoantibodies (Abs) in sera of patients with autoimmune thyroid disease (AITD) and thyroid autoantibody carriers without diagnosed AITD. **Methods:** A hydrogel microarray-based multiplex assay with the chaotrope destruction stage was developed to measure the avidity of thyroid disease-associated autoantibodies, including those targeting thyroperoxidase (TPO), thyroglobulin (Tg), and other minor antigens. **Results:** Evaluation of the assay in three independent cohorts of patients, totaling 266 individuals with and without AITD, demonstrated the heterogeneous avidity of autoantibodies to thyroid proteins. For the confirmation study, the median avidity index (AI) for AbTg was 29.9% in healthy autoantibody carriers, 52.6% for AITD patients, and 92.7% for type 1 diabetes (T1D) thyroid autoantibody carriers. The median AI for AbTPO was 39.9% in healthy carriers, 73.4% in AITD patients, 83.2% in T1D thyroid autoantibody carriers, and 98.5% in AITD patients with thyroid neoplasm. In patients with Hashimoto’s thyroiditis and known disease duration, changes in the avidity maturation of AbTPO over time were demonstrated. **Conclusions:** Longitudinal studies of TPO- and/or Tg-positive healthy individuals (with an interval of 1–2 years between visits) are needed to evaluate the maturation of autoantibody avidity during the asymptomatic phase and to assess the potential of autoantibody avidity as a prognostic marker for disease development.

## 1. Introduction

Autoimmune thyroid diseases (AITD) are the most frequent autoimmune disorders. Among AITD, Hashimoto’s thyroiditis is the most common, while Graves’ disease is less common [1]. The main autoantibody (Ab) target protein in AITDs is thyroperoxidase (TPO), an enzyme involved in the biosynthesis of thyroid hormones, thyroid hormone precursor thyroglobulin (Tg), and thyroid-stimulating hormone receptor [2,3]. The low molecular weight hormones thyroxine (T4) and 3,5,3′-triiodothyronine (T3), in complex with proteins, may also act as targets for Abs [4]. Autoantibodies can be directed against Na/I symporter, megalin, pendrin (PDS), and carboanhydrase II (CA2) [5,6]. In addition, patients with AITDs have a higher rate of detection of other circulating autoantibodies, suggesting a broader autoimmune dysfunction [7]. Some studies suggest a possible link between autoimmune thyroid disease and thyroid cancer [8]. The overexpression of certain proteins, such as pyruvate kinase (PK), may serve as markers for the development of thyroid neoplasia [9]. Corresponding autoantibodies against tumor-associated antigens may also appear [10].

Autoantibodies can be detected in the serum before the clinical manifestation of the disease. However, the detection of autoantibodies is an auxiliary, but not an absolute, diagnostic criterion, and autoantibodies to major thyroid proteins can often be found in individuals without AITDs [11]. It is unknown whether autoantibody carriers are those who will develop the disease in the future. The prevalence of thyroid Abs in the general population is 8–30% for AbTPO and 5–20% for AbTg [5]. In differentiated thyroid cancer, the proportion of patients with positive AbTg rises to 25% [12]. Increased incidence is seen in other autoimmune diseases, particularly AbTPO prevalence in rheumatoid arthritis—16–37%; in type 1 diabetes mellitus (T1D)—40%; in celiac disease—12–30% and AbTg prevalence in rheumatoid arthritis—12–23%; in T1D—30%; in celiac disease—11–32% [13].

Currently, autoantibodies are thought to consist of a polyclonal mixture with different affinities for the autoantigen [14]. This statement also applies to autoantibodies against the major thyroid proteins TPO and Tg [15]. Polyclonal antibodies, by binding to several different epitopes of an autoantigen, may exhibit heterogeneous binding strengths due to multidimensional affinity interactions. This binding strength is called functional affinity or avidity and is widely used in the diagnosis of infectious diseases to distinguish between primary and chronic forms [16]. The methods used to assess the affinity of monoclonal antibodies, such as surface plasmon resonance and biolayer interferometry, cannot be directly applied to evaluate the functional affinity of polyclonal antibodies in serum [17]. Special methods are required to monitor polyclonal antibody–antigen interactions [18]. Avidity can also be assessed by disrupting the polyclonal antibody–antigen complex under chaotropic conditions using immunoassays [19].

It has now been suggested that in autoimmune diseases, autoantibody avidity may influence their pathogenicity [20]. The majority of studies investigating autoantibody avidity in autoimmune diseases have focused on systemic diseases such as systemic lupus erythematosus, rheumatoid arthritis, and antiphospholipid syndrome [21,22,23,24]. A number of studies have been performed on autoantibody avidity in celiac disease and T1D [25,26,27]. Known published data on autoantibody avidity in AITD are extremely limited [28,29].

The goal of this study was to evaluate the avidity of autoantibodies associated with thyroid disease in the sera of patients diagnosed with AITD and in autoantibody carriers without AITD. Given that the thyroid gland harbors multiple known autoantigens, it seems reasonable to combine the capabilities of multiplex methods for the simultaneous detection of autoantibodies to TPO, TG, PDS, CA2, PK, T3, and T4 thyroid hormones. Additionally, we aimed to introduce a new characteristic for the detected autoantibodies: the determination of the avidity index (AI). In this work, a specialized hydrogel microarray with immobilized proteins was developed, and a multiplex analysis technique was implemented for the simultaneous determination of the AI of autoantibodies against each of the immobilized thyroid antigens and candidate proteins.

## 2. Materials and Methods

### 2.1. Clinical Data and Serum Samples

This study was conducted according to the guidelines of the Declaration of Helsinki and approved by the local ethics committee of the Endocrinology Research Centre, Ministry of Health of Russia, Moscow, Russia (protocol No. 17 and date of approval 27 September 2017, Supplementary File S1). The study enrolled 266 adult patients from three independent cohorts: cohort I (*n* = 117) was designated for the development of the multiplex avidity assay, while cohorts II and III (*n* = 149) were utilized for confirmatory studies. The exclusion criteria for the study included the presence of chronic kidney disease at stage C3b or higher, the use of drugs that affect immune system function (such as interleukins, interferons, immunoglobulins, immunosuppressants, and cytostatics), whether currently or in the past, as well as the use of glucocorticosteroids and enzyme inhibitors (e.g., mitotane, ketoconazole). Serum samples were collected from patients and stored at −80 °C.

Patient characteristics for cohort I are shown in Table 1. The mean age of the 117 patients in the cohort was 52.7 (19–84) years. In total, 102 (87.2%) patients were female and 15 (12.8%) were male. The “AITD” group included patients diagnosed with Hashimoto’s thyroiditis (*n* = 19), Graves’ disease (*n* = 2), and patients with a combination of AIT and T1D (*n* = 7). The group without diagnosed AITD included patients with non-toxic multinodular goiter (*n* = 7), thyroid nodules (*n* = 7), and histologically confirmed papillary and medullary thyroid cancer (*n* = 2). The “healthy” group included patients with no endocrine and autoimmune pathology, according to the survey data. The “AbTPO+” group included AbTPO-positive patients as determined by chemiluminescence analysis without ultrasound data.

Patient characteristics for cohort II are shown in Table 2. The mean age of the 123 patients in the cohort was 41.6 (18–76) years. Of these, 105 (85.4%) patients were female, 18 (14.6%) were male. The “AITD only” group included patients diagnosed with Hashimoto’s thyroiditis (*n* = 11) and Graves’ disease (*n* = 8). The “AITD + T1D” group included patients with a combination of AITD and type 1 diabetes mellitus (*n* = 17). The “AITD + other AID” group included patients with a combination of AITD and Addison’s disease and/or Hirata’s disease and/or vitiligo and/or alopecia areata (*n* = 61). The “AITD + PTC” group included patients with a combination of Hashimoto’s thyroiditis and histologically confirmed papillary thyroid cancer (*n* = 6). Patients with type 1 diabetes mellitus without diagnosed AITD were included in the “T1D” group. The “healthy” group included patients with no endocrine pathology (autoimmune and non-autoimmune), according to survey data.

In cohort III, patients with a known duration of the disease were included (*n* = 26). The mean age of the patients in cohort III was 36.0 years (range: 19–69 years). Twenty patients (76.9%) were female, while six (23.1%) were male. The “AITD“ group consisted of patients with Hashimoto’s thyroiditis, diagnosed between nine months and 29 years ago (*n* = 22). The ’healthy’ group (*n* = 4) included individuals with no endocrine pathology (both autoimmune and non-autoimmune) based on survey data.

### 2.2. Chemiluminescence Immunoassay

The level of AbTPO was determined by chemiluminescent immunoassay on the automated analyzer ARCHITECT i2000 (Abbott, IL, USA). AbTg levels were determined by electrochemiluminescence analysis on the Cobas 6000 automated analyzer (Roche Diagnostics, Mannheim, Germany).

### 2.3. ELISA

Commercially available ELISA kits from Xema Co., Ltd. (Moscow, Russia) for measuring AbTPO and AbTg were used for evaluation according to the manufacturers’ instructions: serum samples were diluted 1:101. A total of 100 µL of diluted samples and calibration samples were added to the wells of a plate coated with the corresponding antigen (TPO or Tg). The wells were incubated for 30 min at 37 °C. After incubation, the wells were washed three times. Next, 100 µL of mouse anti-human IgG-HRP conjugate was added to all wells and incubated for 30 min at 37 °C. The wells were washed five times. Subsequently, 100 µL of tetramethylbenzidine substrate was added to each well and incubated in the dark at room temperature for 15 min. The reaction was stopped by adding 100 µL of stop reagent to the wells. Optical density was measured immediately at a wavelength of 450 nm using a HiPo MPP-96 microplate photometer (Biosan, Riga, Latvia).

### 2.4. ELISA with a Chaotropic Agent

For all samples (Cohort II and III) with positive AbTPO and AbTg, the avidity index was measured by modified ELISA. A chaotropic agent incubation step was added to the manufacturer’s standard protocol after incubation with the samples, and the subsequent steps were performed without modification. Kits from Xema Co., Ltd. (Moscow, Russia) were used. All reagents used were from ELISA kits except for those in the denaturation step (5M urea solution and PBS). Serum samples were diluted 1:101. A total of 100 µL of diluted samples and calibration samples were added to the wells of a plate coated with the corresponding antigen (TPO or Tg). The wells were incubated for 30 min at 37 °C. After incubation, the wells were washed three times. Then, 100 µL of a 5M urea solution (denaturing conditions) or PBS (control) was added to the respective wells and incubated for 5 min at 37 °C, followed by three washes. Next, 100 µL of mouse anti-human IgG-HRP conjugate was added to all wells and incubated for 30 min at 37 °C. The wells were washed five times. Subsequently, 100 µL of tetramethylbenzidine substrate was added to each well and incubated in the dark at room temperature for 15 min. The reaction was stopped by adding 100 µL of stop reagent to the wells. Optical density was measured immediately at a wavelength of 450 nm using a HiPo MPP-96 microplate photometer (Biosan, Riga, Latvia).

### 2.5. Microarray Manufacturing

Molecular profiling of autoantibodies in serum samples was performed using hydrogel-based low-density microarrays [30]. All proteins were spotted on each microarray slide in quadruplicate. The following proteins were used: TPO and Tg #8TG52 (HyTest, Turku, Finland), Tg # R132, T3-HRP, and T4-HRP (Xema Co., Ltd., Moscow, Russia), PDS, PK, and CA2 (Cusabio, Wuhan, China) (Figure S1). In addition, the microarray structure included empty elements without immobilized proteins in eight replicates, marker elements and control elements for antibody detection and control of interference of high concentrations of autoantigens in serum (titration of human IgG (Athens Research and Technology, Athens, GA, USA), and mouse monoclonal Abs against autoantigens (mAb Tg, mAb TPO, mAb T4, and mAb T3 (HyTest, Turku, Finland)). Microarrays were manufactured in accordance with the ISO 13485:2016 (https://www.iso.org/obp/ui/#iso:std:iso:13485:ed-3:v1:en, accessed on 10 January 2024) quality management systems (EIMB certificate No. 01.RU.01.21.2031, valid until 28 December 2027). Two-stage quality control was applied to each microarray batch. First, technical quality control of the microarray elements was conducted using specialized optics and computer image analysis. Microarrays with deviations in the geometric parameters of elements not exceeding 10%, as well as deviations in microarray parameters between batches not exceeding 20%, were used for further testing. At the next stage, biological quality control was performed using AutoQon AT controls (Xema Co., Ltd., Moscow, Russia), prepared from patient sera and plasma containing various levels of autoantibodies to Tg and TPO. The presence of specific signals in the microarray elements with immobilized Tg and TPO was confirmed for at least eight microarrays in each batch. Batches of microarrays that passed the two-stage quality control were used in further experiments.

### 2.6. Modified Multiplex Assay

The assay was performed as described previously [31] with minor modifications. To better differentiate between specific and nonspecific interactions, the incubation step with the sample was shortened, while the detection step was extended. To evaluate the avidity index, patient serum samples were assayed in duplicate. Samples were diluted 1:100 and applied on two microarrays (100 µL on each) from the same batch that had passed quality control. After incubation with the sample (for two hours, 37 °C), intermediate washing (PBS with 0.1% Tween 20, 20 min), rinsing, and drying, 5M urea solution (denaturing conditions) or PBS (control) was applied to the corresponding microarrays, incubated for 10 min, and then washed (PBS with 0.1% Tween 20, 20 min). The binding was revealed with anti-human IgG-Cy5.5 (2.5 μg/mL; 50 μL). After overnight incubation at 37 °C, the microarrays were washed (PBS with 0.1% Tween 20, 30 min), rinsed, and dried by centrifugation.

To select the optimal concentration of urea, AutoQon AT controls were used.

### 2.7. Analysis of Fluorescence and Interpretation of Results

Fluorescence images of microarrays were obtained using a proprietary laser-excited microarray analyzer [32]. Fluorescence signals were calculated using proprietary software. For each group of n elements containing the same antigens, the resulting I_n_ signal value was calculated as the median of the four corresponding fluorescence signals. To account for the influence of the total IgG concentration on the result of the Abs assay, the individual background signal from empty gel elements (I_n_/I_ref_) was considered in the analysis for each sample. Specific signals from the element with immobilized antigen I_n_ that were at least two times greater than the individual background signal of I_ref_ were considered positive. All microarray slides were also scanned using a GenePix 4100A microarray reader (Molecular Devices, CA, USA) equipped with GenePix Pro microarray analysis software. No differences were found in the detection of positive signals from microarray elements when using the two scanning systems.

### 2.8. Avidity Index of Autoantibodies

Only samples with positive autoantibodies were included in the calculation of the index. The avidity index for the multiplex immunoassay was calculated by taking the ratio of the fluorescence signals from the elements with immobilized antigen in the denaturing agent-treated sample to those in the immunoassay buffer-treated sample and then multiplying that ratio by one hundred. For ELISA analysis, the avidity index was calculated similarly, using the optical densities of microplate wells that were either treated or untreated with the denaturing agent.

### 2.9. Statistical Analysis and Data Presentation

Statistical analysis was performed using MedCalc (Ostend, Belgium). Pairwise comparisons within groups were conducted using the Mann–Whitney test. Statistical significance was considered at *p* < 0.05. A heat map of normalized fluorescent signals from microarray elements was generated using the Galaxy server (https://usegalaxy.org/, accessed on 8 May 2023).

## 3. Results

### 3.1. Development of the Multiplex Avidity Assay (Cohort I)

**Figure 1:**
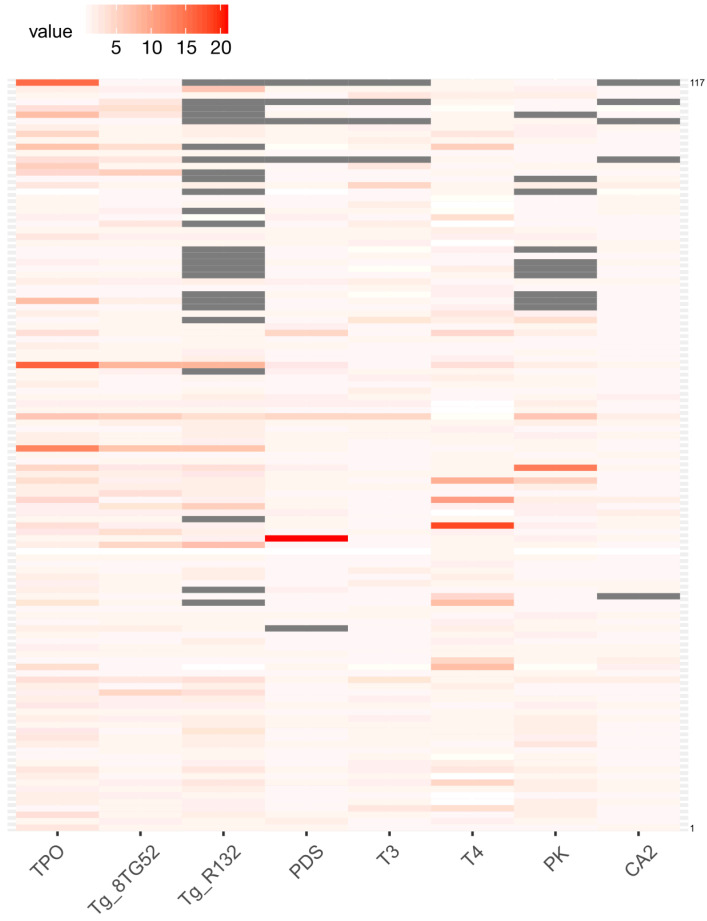
Heat map displaying the fluorescent signals obtained from microarray elements with immobilized autoantigens after sample analysis (*n* = 117). The signals are normalized and scaled by row.

A microarray with immobilized autoantigens TPO, Tg (two proteins from different manufacturers), PDS, CA2, PK, and thyroid hormones T3 and T4 conjugated to horseradish peroxidase was constructed according to a previously developed and validated method for multiplex autoantibody assay [31] (Figure S1). A total of 117 patient serum samples from cohort I were examined using a multiplex platform: AITD patients (*n* = 28), patients with non-autoimmune thyroid diseases (*n* = 16), patients with no endocrine pathology, whether autoimmune or not (*n* = 31), and AbTPO+ patients without thyroid ultrasound investigation (*n* = 42) were included in the study. The multiplex immunoassay revealed that the highest quantity of positive autoantibody signals was obtained from immobilized TPO, Tg, and T4 antigens (Figure 1). For all other antigens, single samples with positive autoantibodies were identified. The results of the multiplex method showed the following frequencies of autoantibodies to different proteins for cohort I: AbTPO 35.0% (41/117); AbTg 23.1% (27/117); AbPDS 3.4% (4/117); AbT3 6.8% (8/117); AbT4 13.7% (16/117); AbCA2 0.85% (1/117); and AbPK 5.1% (6/117).

A modified multiplex method was proposed to determine the avidity index of autoantibodies. Three experimental assay schemes were tested. The first involved the destruction of weak interactions by a denaturing agent after the formation of the antigen–Ab complex. The second involved the addition of the denaturing agent during the incubation with the sample, which would avoid adding an additional denaturation step to the analysis. The third approach was based on adding the agent after the formation of the triple “Antigen–Ab–anti-human Ab” complex. This would allow the use of a single microarray for a patient—both as a control and after treatment under denaturing conditions. Five different denaturants were tested: urea (0.25–10 M), thiourea (0.5–10 M), guanidine thiocyanate (0.5–2 M), sodium chloride (0.4–1 M), and polyethylene glycol (20–80% in phosphate-buffered saline (PBS)). PBS was used to analyze the sample under control (non-denaturing) conditions. NaCl and PEG had no effect within the selected range. For thiourea and guanidine, microarrays exposed to these agents did not yield clean fluorescent images for all samples. Thus, the use of these buffers can lead to fluorescent artifacts. When the sample was tested repeatedly, the use of urea after the formation of the antigen–Ab complex (the first scheme) gave the most reproducible results, with a coefficient of variation for AI of less than 15%. To select the optimal concentration of urea for the first scheme in the microarray-based avidity assay, a series of experiments was conducted. The fluorescence intensity varied only slightly with urea concentrations ranging from 0 to 4 M but began to decrease at 5 M for both immobilized TPO and Tg when analyzed using AutoQon AT controls (pooled patient sera) (Figure S2). Consequently, 5 M urea was selected as the chaotropic agent for the modified multiplex method.

**Figure 2:**
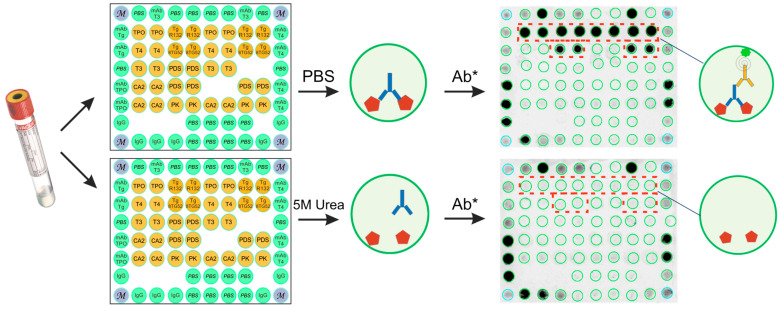
Multiplex autoantibody avidity assay. Serum sample is added to two microarrays with immobilized autoantigens and incubated to capture the target autoantibodies. Following this, one microarray was incubated with 5 M urea for 10 min, while the other was incubated with a blank buffer (PBS—phosphate-buffered saline). Autoantibody binding is detected using fluorescently labeled anti-human IgG (Ab*). The designation of the probes immobilized on the microarray is shown in Figure S1.

Figure 2 presents a graphical representation of the modified multiplex assay and fluorescence images of hydrogel microarray after sample assay, both with and without 5 M urea treatment. Treatment with the chaotropic agent removes low-avidity antibodies from the resulting antigen–Ab complex. Thus, one microarray detects all autoantibodies in the sample, while the second microarray detects only high-avidity autoantibodies. The measurement of avidity is expressed as the avidity index (AI, %), calculated by the ratio of fluorescence intensity from the corresponding immobilized antigen after treatment with a denaturing agent compared to the untreated sample.

**Figure 3:**
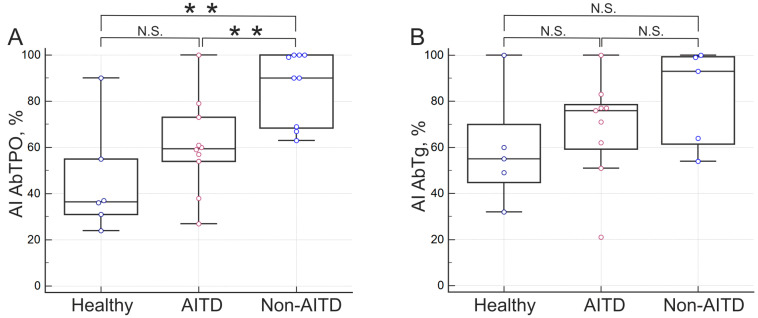
Avidity indexes (%) for different patient groups: (**A**) for AbTPO, (**B**) for AbTg. Abbreviations: AITD—autoimmune thyroid disorders; non-AITD—group of patients without diagnosed AITD. ** *p* < 0.01; N.S., not significant (*p* ≥ 0.05).

Avidity indexes were calculated for samples positive for at least one autoantibody in the multiplex assay. AI autoantibodies in patient groups with confirmed diagnoses from cohort I (Figure 3) were compared in terms of AI AbTPO (*n* = 25) and AI AbTg (*n* = 19). For other antigens, avidity indexes were determined for individual samples with positive Ab (total *n* = 19); however, the outcomes showed significant variation.

### 3.2. Confirmation Studies with Independent Patient Cohort (Cohort II)

Serum samples from 113 patients (*n* = 103 with AITD, *n* = 10 with T1D) and ten healthy donors from independent cohort II were analyzed using the multiplex avidity assay. Subgroups of AITD patients were identified, including those with isolated AITD (*n* = 19), AITD and T1D (*n* = 17), AITD and other endocrine and comorbid autoimmune diseases (*n* = 61), and AITD and papillary thyroid carcinoma (*n* = 6).

The frequencies of autoantibodies targeting different proteins in cohort II, as determined by the multiplex method, were as follows: AbTPO 68.3% (84/123), AbTg 35.8% (44/123), AbPDS 0% (0/123), AbT3 1.6% (2/123), AbT4 8.1% (10/123), AbCA2 0.8% (1/123), AbPK 1.6% (2/123). Avidity indexes were calculated in a multiplex assay for samples positive for at least one autoantibody. Avidity indexes were also measured for AbTPO- and AbTg-positive samples using a modified enzyme-linked immunosorbent assay (ELISA) by including an additional incubation step with 5M urea or blank buffer to the standard protocol. In cohort II patients, AbTPO and AbTg levels were also measured by chemiluminescent immunoassay (CLIA).

**Figure 4:**
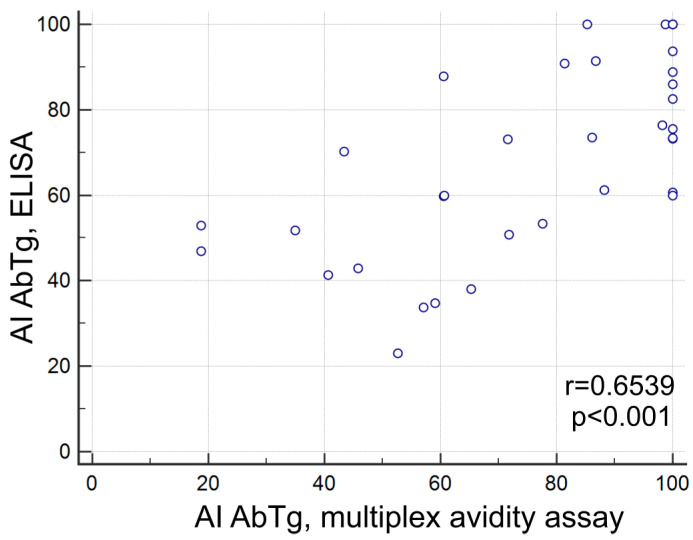
Autoantibody avidity index (AI, %) in multiplex avidity assay and ELISA for AbTg (r = 0.6539, *p* < 0.001, *n* = 34).

A comparison of AbTg detection results using different methods (multiplex avidity assay, CLIA, and ELISA) revealed a satisfactory correlation. The correlation between CLIA and multiplex avidity assay measurements was r = 0.6412 (*p* < 0.0001, *n* = 121), while the correlation between ELISA and multiplex avidity assay measurements was r = 0.6417 (*p* < 0.0001, *n* = 44). The correlation between the CLIA and ELISA measurements was r = 0.6286 (*p* < 0.0001, *n* = 42). A correlation was found between the AbTg avidity score assessed by multiplex avidity assay and ELISA, with a positive correlation coefficient of r = 0.6539, *p* < 0.001, and a sample size of *n* = 34 (Figure 4).

**Figure 5:**
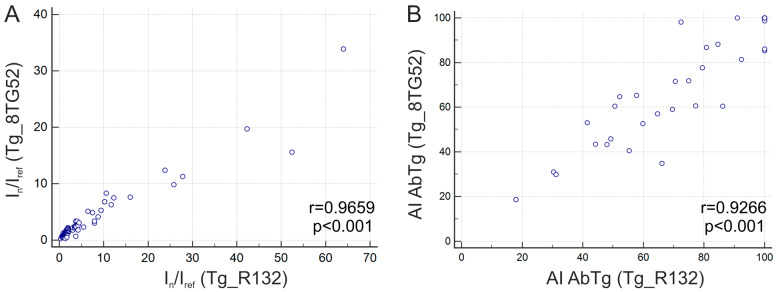
Comparison of serum sample results for two different immobilized Tg preparations (8TG52 and R132): (**A**) normalized fluorescence signals I_n_/I_ref_ obtained from the respective immobilized Tg preparations (r = 0.9659, *p* < 0.001, *n* = 123); (**B**) avidity indexes (AI, %) calculated for positive results (r = 0.9266, *p* < 0.001, *n* = 41).

Tg antigens from two different suppliers (Hytest, Finland and Xema Co., Ltd., Moscow, Russia) were used for immobilization on the microarray. The serum sample analysis presented excellent agreement among fluorescence signals obtained from various immobilized preparations (r = 0.9659, *p* < 0.001, *n* = 123) and high agreement when measuring the avidity index of AbTg (r = 0.9266, *p* < 0.001, *n* = 41) (Figure 5).

The comparison of AbTPO detection results using three methods demonstrated a satisfactory correlation. The CLIA and multiplex avidity assay measurements showed an r-value of 0.6255 (*p* < 0.0001, *n* = 121). ELISA and multiplex avidity assay measurements had an r-value of 0.5795 (*p* < 0.0001, *n* = 44). Additionally, the CLIA and ELISA measurements yielded an r-value of 0.6286 (*p* < 0.0001, *n* = 42). However, the results for AI AbTPO obtained using two methods (ELISA and multiplex) did not correlate. There was no correlation found between the level of fluorescent signals and AI for either AbTg or AbTPO. For the other antigens (PDS, T3, T4, CA2, PK), single samples with positive autoantibodies were identified; the AI varied in a wide range (23.3–100%).

**Figure 6:**
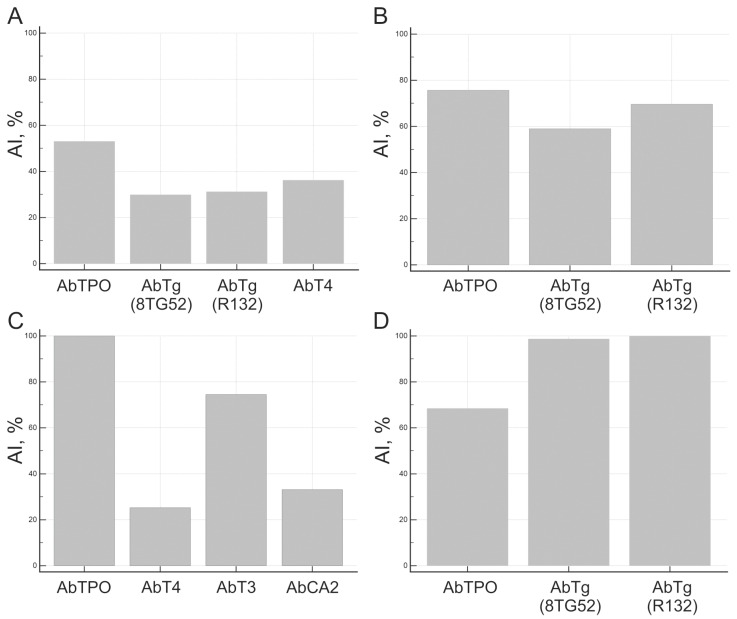
Avidity index of autoantibodies to different thyroid antigens for individual patients: (**A**) healthy donor; (**B**) Hashimoto’s thyroiditis; (**C**) Grave’s disease, and (**D**) type 1 diabetes mellitus. The coefficient of variation for AI is less than 15%.

Differences in autoantibody avidity were studied and found to be personalized to each patient for various thyroid antigens. As an example, Figure 6 displays the avidity indexes of serum samples analyzed from healthy thyroid autoantibody carriers (A), AITD patients (B, C) and T1D thyroid autoantibody carriers (D).

**Figure 7:**
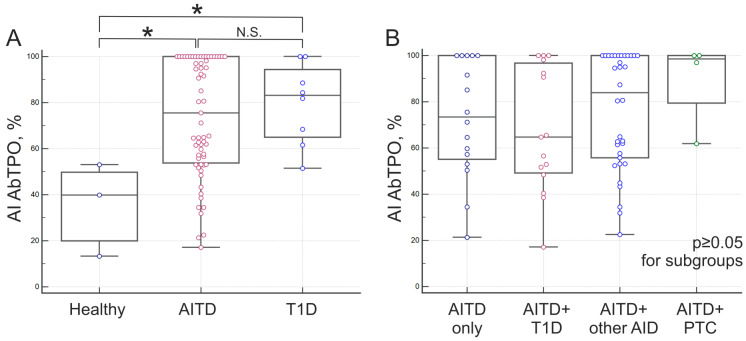
Avidity of autoantibodies to TPO: (**A**)—for groups of patients with autoimmune thyroid disease (AITD), autoantibody carriers—healthy and patients with type 1 diabetes mellitus (T1D); (**B**)—distribution of AI AbTPO within the group of patients with AITD: isolated AITD (AITD only), AITD combined with T1D (AITD + T1D), AITD combined with other endocrine and comorbid autoimmune diseases excluding T1D (AITD + other AID), and patients with papillary thyroid carcinoma and AITD (AITD + PTC). * *p* < 0.05; N.S., not significant (*p* ≥ 0.05).

AI was calculated for AbTPO-positive samples from 79 AITD patients and 11 autoantibody carriers from T1D patient groups and healthy donors. The median AI AbTPO was 39.9% for healthy autoantibody carriers, 73.4% for patients with isolated AITD, 64.8–80.6% for patients with other AID combined with AITD, 98.5% for patients with papillary thyroid carcinoma combined with AITD, and 83.2% for patients of autoantibody carriers with type 1 diabetes (T1D) (Figure 7). The median AI AbTPO difference between the presented groups was found to be statistically significant in the AITD/healthy (*p* = 0.0223) and T1D/healthy (*p* = 0.0247) groups, but not in the AITD/T1D groups (*p* ≥ 0.05) (Figure 7A). Patients with AITD were divided into subgroups based on isolated AITD (AITD only), AITD combined with other autoimmune endocrine and comorbid pathologies (AITD + other AID; AITD + T1D), or thyroid autoimmunity combined with papillary carcinoma (AITD + PTC). There were no significant differences in the medians of AI Ab TPO for these subgroups (*p* ≥ 0.05) (Figure 7B).

**Figure 8:**
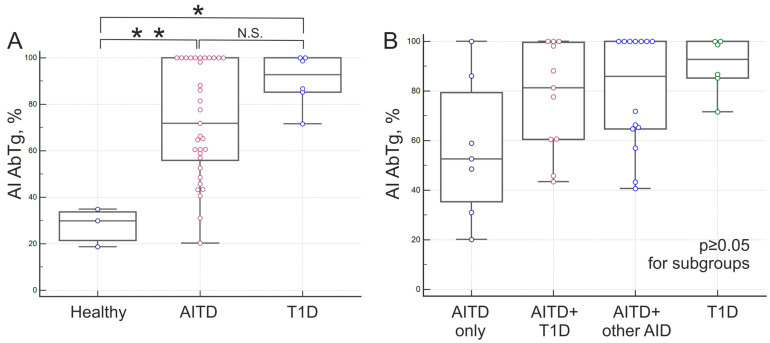
Avidity of autoantibodies to Tg: (**A**)—for autoimmune thyroid disease (AITD) patient groups, autoAT carriers—healthy and patients with type 1 diabetes mellitus (T1D); and (**B**) distribution of AI AbTg among the AITD patient group, which includes isolated AITD (AITD only), AITD combined with other endocrine and comorbid AIDs except for type I diabetes mellitus (AIT + other AIDs), and AITD combined with type I diabetes mellitus (AITD + T1D). ** *p* < 0.01; * *p* < 0.05; N.S., not significant (*p* ≥ 0.05).

AI values were obtained for AbTg-positive samples from patients with AITD (*n* = 35) and autoantibody carriers (*n* = 9) from the T1D patient groups and healthy donors. According to Figure 8, the median AI values for AbTg were 29.9% in healthy autoantibody carriers, 52.6% in patients with isolated AITD, 81.4–85.9% in patients with combined AITD and other AIDs, and 92.7% in autoantibody-positive patients with T1D. The medians of AI AbTg in the presented groups showed statistically significant differences for healthy/AITD (*p* = 0.0071) and healthy/T1D (*p* = 0.0201) groups (Figure 8A), but not for AITD/T1D groups (*p* ≥ 0.05). Median AI AbTg differences among subgroups of AITD patients were statistically insignificant (*p* ≥ 0.05) (Figure 8B). The presence of high-avidity autoantibodies to Tg in patients with T1D was also confirmed by a modified ELISA.

### 3.3. The Impact of Disease Duration on the Avidity of Autoantibodies (Cohort III)

Serum samples from patients with Hashimoto’s thyroiditis with known disease durations (*n* = 22) and four healthy donors from an independent cohort (Cohort III) were analyzed using multiplex avidity assays, CLIA, and ELISA. The comparison of AbTPO detection results using three methods demonstrated a satisfactory correlation. The measurements from the CLIA and multiplex avidity assay showed an r-value of 0.7831 (*p* < 0.0001, *n* = 26). The ELISA and multiplex avidity assay yielded an r-value of 0.7172 (*p* < 0.0001, *n* = 26). Additionally, the CLIA and ELISA measurements produced an r-value of 0.6791 (*p* < 0.0001, *n* = 26). The comparison of AbTg detection results using three methods also demonstrated a satisfactory correlation. The measurements from the CLIA and multiplex avidity assay showed an r-value of 0.8932 (*p* < 0.0001, *n* = 26). The ELISA and multiplex avidity assay yielded an r-value of 0.7115 (*p* < 0.0001, *n* = 26). Additionally, the CLIA and ELISA measurements produced an r-value of 0.7782 (*p* < 0.0001, *n* = 26).

**Figure 9:**
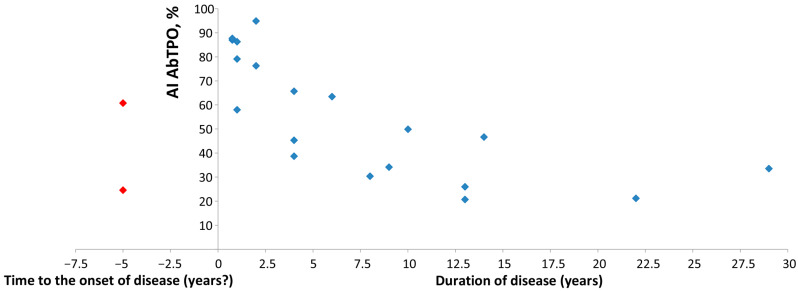
Avidity of autoantibodies to TPO in Hashimoto’s thyroiditis patients with known disease duration (from nine months to 29 years, blue dots) and two healthy carriers (time to the onset of disease is unknown, red dots). Data were confirmed by modified ELISA for AbTPO-positive samples.

Avidity index (AI) was evaluated using the multiplex avidity assay and modified ELISA for AbTPO-positive samples from 19 Hashimoto’s thyroiditis patients and two healthy controls, as well as for AbTg-positive samples from five Hashimoto’s thyroiditis patients and two healthy controls. The association between AI for AbTPO and disease duration was assessed, and the data were subsequently validated through the use of a modified enzyme immunoassay employing 5 M urea as a chaotropic agent (Figure 9).

## 4. Discussion

Currently, antibody avidity assessment is used to evaluate the immune response after vaccination and to differentiate acute and chronic viral infection. A modified ELISA with a chaotropic agent is the most commonly used method for assessing antibody avidity due to its simplicity and availability [16]. In addition to ELISA, further studies are investigating the use of multiplex assays to quantify antibody avidity in viral infections [33,34]. The modified multiplex assay presented in this study is based on indirect autoantibody detection. The methodology involves covalent copolymerization immobilization of antigens in the hydrogel elements of a microarray [30]. Next, a blood serum sample is incubated on the microarray, and fluorescently labeled anti-IgG antibodies are used to detect autoantibodies in the sample [31]. In the modified assay, two microarrays are used, with a chaotropic agent incubated on one microarray after the serum sample. The hydrogel microarray element contains covalently immobilized proteins, and exposure to a chaotrope selectively releases autoantibodies bound to the immobilized antigen. This procedure enables the exclusive detection of high-affinity autoantibodies. No chaotropic treatment is applied to the control microarray. The autoantibody avidity index is the percentage of Abs that remain bound to the immobilized antigen after chaotrope treatment.

Resistance to chaotropic agents can evaluate antibody avidity in two ways: elution and dilution [16]. The elution principle involves the addition of a chaotropic agent after the formation of autoantibody–antigen complexes to disrupt weak interactions. In contrast, the dilution principle involves adding the chaotropic agent to the sample prior to the assay to prevent the formation of these complexes. Avidity can be measured by the degree of IgG release from its antigen following treatment with a chaotropic agent, such as urea [35]. Other agents that disrupt the binary complex can also be used, with varying effectiveness depending on the specific antigen [36]. For example, it has been shown that 6–8 M urea is required to determine the avidity index of anti-phospholipid autoantibodies [23]. The optimal method, which involves eluting antibodies from autoantibody–antigen complexes, and the optimal chaotropic agent (5 M urea) were selected in the current study based on results showing the most consistent outcomes in repeated sample analyses.

The optimal assay scheme was tested using sera from cohort I patients (*n* = 117). AIs were calculated for positive samples of at least one autoantibody. In the present study, no correlation was observed between the fluorescence signal levels and the AI of autoantibodies. This can be attributed to the principle of the method. The indirect ELISA and multiplex methods detect the degree of occupation of immobilized antigen autoantibodies. However, the detectable antigen occupancy is influenced not only by antibody concentration but also by affinity. The same level of occupancy can be achieved by increasing the concentration of low-affinity antibodies and decreasing the concentration of high-affinity antibodies [35]. However, Sliva et al. noticed a correlation between AbTPO levels and AI in a previous study with a correlation coefficient of 0.444 and a *p*-value of 0.008 [28]. Other researchers have also found no correlation between the level and avidity of autoantibodies with respect to anti-beta2-glycoprotein I antibodies [36] and anti-cardiolipin autoantibodies [23].

When analyzing AI AbTPO and AI AbTg for cohort I using a small subset of positive samples from patients with known diagnoses, a trend toward increased antibody avidity was observed in the row “healthy-patients with AITD-patients with non-autoimmune thyroid disease”, which included patients with thyroid nodules and malignant thyroid neoplasms. However, the significance of these preliminary findings was limited by the small number of positive samples from autoantibody carrier patients, so a series of confirmatory studies with independent patient cohorts was conducted.

In cohort II of the present study, autoantibodies to the major thyroid autoantigens, TPO and Tg, were additionally analyzed using the enzyme-linked immunosorbent assay and chemiluminescent assay. Autoantibody measurements tend to exhibit greater imprecision compared to those observed in clinical chemistry and hematology [37]. The detection of antibody levels to thyroid antigens, including both TG and TPO, is no exception, exhibiting considerable inter-method variability, especially for high-level Abs samples. This may be due to differences in the proteins used for immobilization, which can affect the exposure of immunodominant epitopes recognized by the polyclonal antibodies in serum, as well as potential interference from elevated antigen concentrations, particularly TG, in the patients’ sera [3]. Other factors include differences in assay methodology and sensitivities, as well as variations in detection antibodies. To exclude the influence of the measurement method on the avidity index assay results, only samples that were positive by all three methods were included in the autoantibody avidity measurement. For triple-positive samples, avidity measurements were conducted using modified mono- (ELISA) and multiplex (protein microarray) assays.

Comparison of fluorescence intensity (multiplex) with the corresponding CLIA and ELISA units revealed a satisfactory correlation between the three methods for AbTg: r = 0.6412, *p* < 0.0001, *n* = 121 (CLIA/multiplex); r = 0.6417, *p* < 0.0001, *n* = 44 (ELISA/multiplex); r = 0.6760, *p* < 0.0001, *n* = 42 (CLIA/ELISA). Moreover, a positive correlation was observed between AI AbTg measured by multiplex and ELISA, which were similarly modified (5 M urea treatment). The multiplex method for measuring avidity offers the advantage of selectively targeting the bonding in the binary complex formed between autoantigen and autoantibody through treatment with a denaturing agent. This ensures that the immobilized antigen remains securely covalently bound in the hydrogel. In contrast, passive adsorption of the protein onto the microplate plastic in ELISA can lead to the detachment of the autoantigen from the solid phase when exposed to a denaturing agent. However, the comparison of two modified methods, ELISA and multiplex, revealed an agreement for AI AbTg (r = 0.6539; *p* < 0.001, *n* = 34), which was not observed for AI AbTPO. Notably, significant discrepancies were observed in the absolute quantification of autoantibody titers. The correlations for AbTPO between the three methods were as follows: r = 0.6255, *p* < 0.0001, *n* = 121 (CLIA/multiplex); r = 0.5795, *p* < 0.0001, *n* = 44 (ELISA/multiplex); r = 0.6286, *p* < 0.0001, *n* = 42 (CLIA/ELISA). As for cohort I, no correlation was found between fluorescence signal levels and AI for either AbTg or AbTPO. To assess the impact of different immobilized autoantigen preparations on the level of measured autoantibody avidity, two different Tg proteins from diverse sources were incorporated into the microarray structure. The AI for AbTg remained consistent regardless of the recombinant proteins used for immobilization (r = 0.9266, *p* < 0.001, *n* = 41).

In this study, we assessed the avidity of various autoantibodies in a single assay for individual patients. Previous research has highlighted the challenge of comparing results across studies, owing to the lack of a standardized method for detecting antibody avidity and the absence of a uniform classification for high- and low-avidity antibodies [16]. The multiplex assay offers an advantage in determining the avidity of different autoantibodies under the same conditions in a single experiment. Consistent assay conditions allow for measuring the variation in the avidity of autoantibodies to different proteins in a single patient. It has been shown that an individual can have both high- and low-avidity autoantibodies to various proteins in thyroid tissue. Additionally, differences in median values were observed between the AITD/healthy and T1D/healthy groups for individuals with antibodies. The AI AbTPO exhibited considerable variability within the AITD group. Further subgrouping of AITD patients based on isolated AITD (AITD only), AITD combined with other autoimmune endocrine and comorbid pathologies (AITD + other AID; AITD + T1D), or AITD combination of thyroid autoimmunity with papillary carcinoma (AITD + PTC) did not reveal statistically significant differences in median AI AbTPO. Similar findings were reported by Silva et al., who observed comparable AI AbTPO levels in patients with subclinical (sH) and overt hypothyroidism (H), despite differing medians between the groups [28]. The median AI AbTPO for group H was 72.5% (66.75–78.25), and for sH, it was 48.05% (35.4–63.35). Meanwhile, the healthy control group had a median of 34.53% (32.76–36.30), despite the authors encountering a similar issue of a limited number of autoantibodies carriers among the investigated healthy control group (only three AbTPO+ patients).

Statistically significant differences in AI AbTg medians were observed when comparing positive samples from AITD patients and autoantibody carriers. Differences in AI medians were also noted between the healthy/AITD and healthy/T1D groups. Further categorization of AITD patients into subgroups revealed significant differences in AI AbTg medians (*p* = 0.0452) between patients with T1D and those with isolated AITD without comorbid pathologies. All samples from T1D patients with high avidity AbTg detected by the multiplex method also exhibited high avidity when assessed using a modified ELISA. In a prior study, Zhang et al. examined AbTg avidity in cohorts of patients with Hashimoto’s thyroiditis, finding reduced avidity in patients in a state of euthyroidism compared to those with subclinical hypothyroidism and hypothyroidism [29]. However, it was not possible to compare the results, as none of the studied patients had other autoimmune diseases and there was no group of autoantibody carriers without AITD. We do not exclude the possibility that, for the large thyroglobulin, autoantibodies in patients with AITD and T1D may interact with different epitopes, whereas for thyroperoxidase, they may interact with the same epitope. This interaction could explain the difference in the avidity index for Tg (T1D/AITD) but not for TPO (T1D/AITD). This most likely accounts for the high AI values in samples from patients with papillary thyroid carcinoma, although our study sample is too small to draw definitive conclusions. The specificity of Ab against different epitopes in different diseases has been demonstrated, as seen, for example, with GAD65 in T1D, autoimmune polyendocrine syndrome type 1, and neurological syndromes [38].

In light of the findings from the study by Silva et al. [28], which demonstrated an inverse correlation between the level of free T4 and the avidity of AbTPO, the methodology we developed has the potential to be used for assessing the risk of developing hypothyroidism in patients without diagnosed AITD who were positive for AbTPO. Furthermore, the results obtained by Zhang et al. [29] regarding AbTg also indicate the feasibility of employing this marker in clinical practice.

Similar to the approach that relies on paired sera to detect changes in antibody avidity against viruses, autoantibody avidity analysis can also track changes in the avidity index of a patient over time. In patients with known disease durations (ranging from 9 months to 29 years) of Hashimoto’s thyroiditis (*n* = 22), the developed multiplex avidity method revealed a correlation between the avidity index of autoantibodies to TPO and disease duration. The disease duration may differ from what is known clinically, as Hashimoto’s thyroiditis often progresses slowly over an extended period, sometimes spanning several years. It is not uncommon for the signs and symptoms of the disease to go unnoticed. Nevertheless, based on the data obtained, it can be inferred that during the latent period of the disease, there is an increase in the avidity of autoantibodies. Similar data were previously reported for anti-citrullinated protein antibodies [39]. The increase and subsequent decrease in autoantibody avidity as the disease progresses may be linked to both immune activation and tissue destruction, as well as changes in the level of autoantigens available to the immune system. The dependence of autoantibody avidity on disease duration is an important parameter for understanding the dynamics of the autoimmune process and may be utilized in the future to develop personalized prognostic approaches.

This study has some limitations. First, the sample size of patients without diagnosed AITD who were positive for AbTPO and/or AbTg was small, which may have influenced the results. Second, the lack of data on disease duration for patients with AITD in cohort I and cohort II may affect the conclusions of the research. Lastly, our proposed multiplex and ELISA immunoassay methodologies for assessing the avidity of thyroid autoantibodies require further interlaboratory standardization and validation.

## 5. Conclusions

The avidity of autoantibodies against thyroid proteins varies over a wide range. However, a trend toward an elevated avidity index can be observed in the sequence: ’healthy subjects > patients with AITD > patients without diagnosed AITD’. These findings, along with the observed changes in avidity maturation of AbTPO over time, suggest the potential use of autoantibody avidity as an additional parameter when analyzing individual patient samples over time. Further investigation is needed to determine whether the detection of both low-avidity autoantibodies in healthy carriers and high-avidity autoantibodies to thyroid proteins in patients without diagnosed AITD or non-autoimmune thyroid pathology is a general phenomenon.

## Figures and Tables

**Table 1:** Patient characteristics of cohort I.

Group	*n*	Mean Age (Range)	Female	Male
AITD	28	54.5 (19–84)	24 (85.7%)	4 (14.3%)
Patients without diagnosed AITD	16	52.7 (24–74)	15 (93.75%)	1 (6.25%)
Healthy	31	51.0 (21–68)	24 (77.4%)	7 (22.6%)
AbTPO+	42	53.9 (21–81)	39 (92.9%)	3 (7.1%)
Total	117	52.7 (19–84)	102 (87.2%)	15 (12.8%)

Abbreviations: AITD—autoimmune thyroid disorders.

**Table 2:** Patient characteristics of the cohort II.

Group	*n*	Mean Age (Range)	Female	Male
AITD only	19	45.4 (18–76)	18 (94.7%)	1 (5.3%)
AITD + T1D	17	39.4 (20–75)	14 (82.3%)	3 (17.7%)
AITD + others AID	61	41.9 (20–76)	56 (91.8%)	5 (8.2%)
AITD + PTC	6	46.2 (20–69)	5 (83.3%)	1 (16.7%)
T1D	10	34.3 (19–45)	5 (50%)	5 (50%)
Healthy	10	43.7 (22–66)	8 (80%)	2 (20%)
Total	123	41.6 (18–76)	105 (85.4%)	18 (14.6%)

Abbreviations: AITD—autoimmune thyroid disorders; PTC—papillary thyroid carcinoma; T1D—Type 1 diabetes mellitus.

## Data Availability

The authors confirm that the data supporting the findings of this study are available within the article and/or its Supplementary Materials.

## Supplementary Materials

The following supporting information can be downloaded at: www.mdpi.com/article/10.3390/diagnostics15030341/s1, Figure S1: Microarray layout. Figure S2: Effect of urea on fluorescence intensity of immobilized thyroid peroxidase and thyroglobulin after microarray incubation with serum and subsequent treatment with urea solution. Supplementary File S1: Endocrinology Research Center Local Ethics Committee Approval (original document and direct translation).
