# Supplementary material for: Microarray-Based Avidity Assay for Assessment of Thyroid Autoantibodies
Source: Diagnostics (Basel). 2025 Jan 31;15(3):341. doi: 10.3390/diagnostics15030341 (PMC11817500; doi:10.3390/diagnostics15030341)
Supplement: Supplementary file 1 [file diagnostics-15-00341-s001.zip › Figure S1 last version.pdf]

M	PBS	mAb T3	PBS	PBS	PBS	PBS	mAb T3	PBS	M
mAb Tg	TPO	TPO	Tg_R132	Tg_R132	TPO	TPO	Tg_R132	Tg_R132	mAb T4
mAb Tg	T4	T4	Tg_8TG52	Tg_8TG52	T4	T4	Tg_8TG52	Tg_8TG52	mAb T4
PBS	T3	T3	PDS	PDS	T3	T3			PBS
mAb TPO	CA2	CA2	PDS	PDS			PDS	PDS	mAb T4
mAb TPO	CA2	CA2	PK	PK	CA2	CA2	PK	PK	mAb T4
IgG				PBS	PBS	PBS	PBS		IgG
M	IgG	IgG	IgG	PBS	PBS	PBS	PBS	IgG	M

Figure S1. Microrray layout. *Abbreviations: TPO - Thyroid peroxidase, Tg – Thyroglobulin, T3-HRP – 3,5,3’-Triiodothyronine (T3) Conjugate with Horseradish Peroxidase, T4-HRP – Thyroxine (T4) conjugate with horseradish peroxidase, PDS - pendrin, PK - pyruvate kinase, CA2 - carbanhydrase 2, IgG – human immunoglobulin G, mAb - mouse monoclonal antibodies, PBS - phosphate-buffered saline (Reference empty elements with no proteins), M - marker elements*
