# Supplementary material for: Microarray-Based Avidity Assay for Assessment of Thyroid Autoantibodies
Source: Diagnostics (Basel). 2025 Jan 31;15(3):341. doi: 10.3390/diagnostics15030341 (PMC11817500; doi:10.3390/diagnostics15030341)
Supplement: Supplementary file 1 [file diagnostics-15-00341-s001.zip › Figure S2 new.pdf]

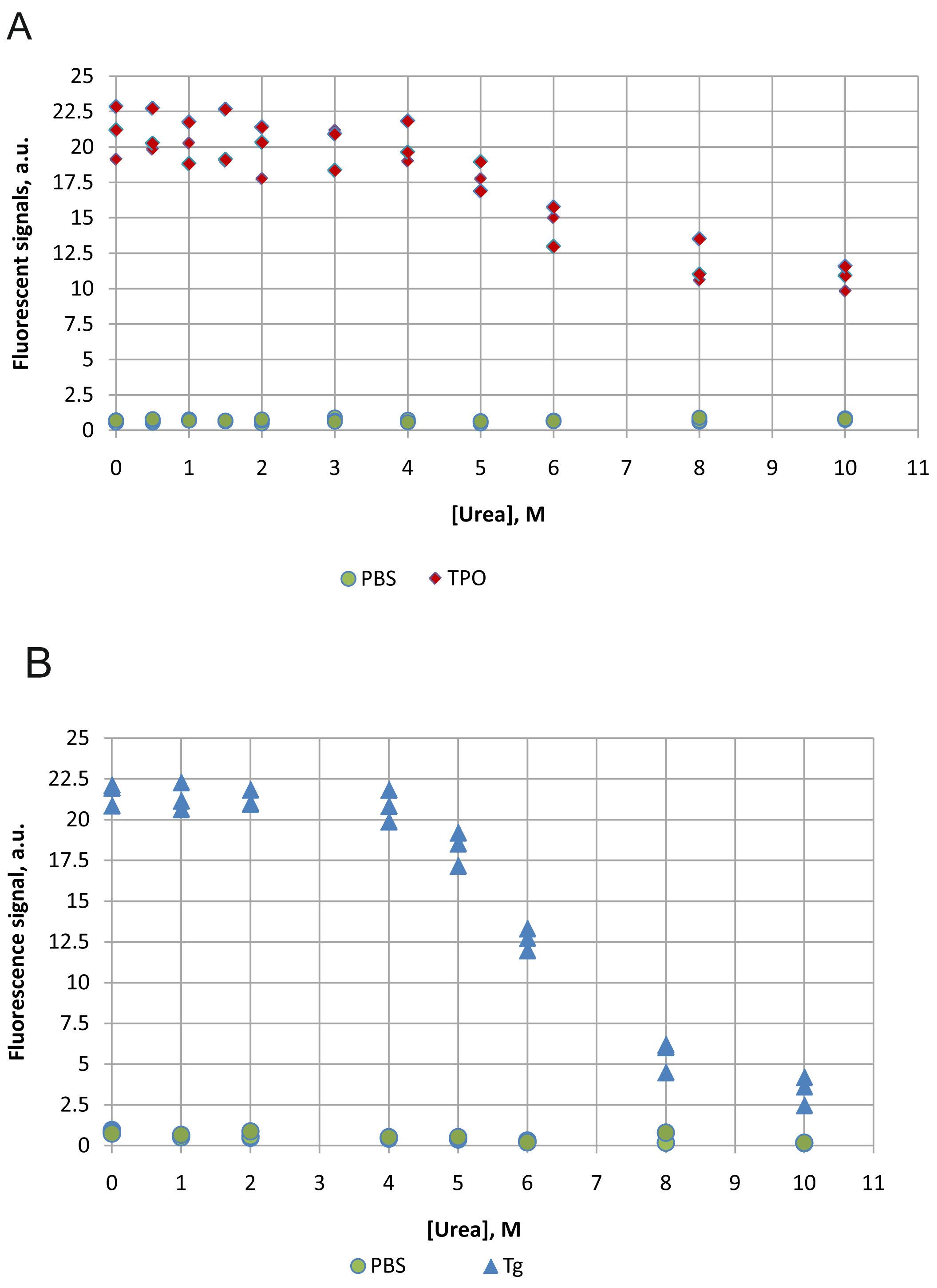


**Figure S2.** The effect of urea on the fluorescence intensity of immobilized TPO antigen–Ab complex **(A)** and Tg antigen–Ab complex **(B)** after microarray incubation with control serum and subsequent treatment with urea solution. AutoQon AT controls (Xema Co., Ltd., Moscow, Russia) prepared from patient's sera and plasma containing various levels of autoantibodies to thyroid antigens (Tg, TPO, and TSH receptor) were used. A pooled sample was analyzed in triplicate. Abbreviations: TPO - Thyroid Peroxidase, Tg - Thyroglobulin, PBS - Phosphate-Buffered Saline (microarray reference elements without proteins)
