# Supplementary material for: Microarray-Based Avidity Assay for Assessment of Thyroid Autoantibodies
Source: Diagnostics (Basel). 2025 Jan 31;15(3):341. doi: 10.3390/diagnostics15030341 (PMC11817500; doi:10.3390/diagnostics15030341)
Supplement: Supplementary file 1 [file diagnostics-15-00341-s001.zip › Supplementary file S1.pdf]

Direct translation

Local Ethics Committee
at the National Clinical Research Centre for Endocrinology
of the Ministry of Health of the Russian Federation
11 Dmitriy Ulyanov str. 11, 117036,
phones: (499) 126-9670, (495) 668-2079 (add. 4809), e-mail: lek_endocrincentr@mail.ru

Extract from protocol No. 17
of the Meetings of the Local Ethics Committee held on 27 September2017

Attended:

Committee Chair:

Andreeva E.N. - doctor of medical sciences, professor, head of the Department of endocrine gynecology of the National Clinical Research Centre for Endocrinology of the Ministry of Health of the Russian Federation.

Deputy Chair of the Committee:

Shestakova M.V. - Academician of the Russian Academy of Sciences, professor, director of the Diabetes Institute of the National Clinical Research Centre for Endocrinology of the Ministry of Health of the Russian Federation.

Secretary of the Committee:

Shatskaya O.A. - candidate of medical sciences, senior researcher of the therapeutic and diagnostic department of functional cardiology of the National Clinical Research Centre for Endocrinology of the Ministry of Health of the Russian Federation.

Committee members:

1. Arapova S.D. - candidate of medical sciences, leading researcher in the Department of neuroendocrinology and osteopathy of the National Clinical Research Centre for Endocrinology of the Ministry of Health of the Russian Federation.
2. Belovalova I.M. - candidate of medical sciences, assistant of the Director of the National Clinical Research Centre for Endocrinology of the Ministry of Health of the Russian Federation.
3. Vedenyapina T.M. - Secretary of the Russian diabetic association of patients.
4. Vikulova O.K. - candidate of medical sciences, leading researcher, head of the Department of epidemiology and state register of diabetes mellitus of the National Clinical Research Centre for Endocrinology of the Ministry of Health of the Russian Federation.
5. Galstyan G.R. - doctor of medical sciences, professor, head of the diabetic foot Department of the National Clinical Research Centre for Endocrinology of the Ministry of Health of the Russian Federation.
6. Zilberman L.I. - candidate of medical sciences, senior researcher of the Department of diabetes mellitus of children and adolescents Department of the National Clinical Research Centre for Endocrinology of the Ministry of Health of the Russian Federation.
7. Zinovieva E.N. - deputy director for economic affairs, National Clinical Research Centre for Endocrinology of the Ministry of Health of the Russian Federation.
8. Melnikova O.G. - candidate of medical sciences, leading researcher of the Diabetes therapy Department with Reference Centre of the National Clinical Research Centre for Endocrinology of the Ministry of Health of the Russian Federation.

9. Shatokhina M.S. - Chief medical nurse of the National Clinical Research Centre for Endocrinology of the Ministry of Health of the Russian Federation.

Considered: On expert examination of the documentation to decide on the approval of the RSCF grant No 17-75-30035 "Autoimmune endocrinopathies with multiorgan lesions: genomic, postgenomic and metabolomic markers. Genetic risk prediction, monitoring, early predictors, personalised correction and rehabilitation" at the National Clinical Research Centre for Endocrinology of the Ministry of Health of the Russian Federation.

Principal Investigator: **Troshina E.A., professor, doctor of medical sciences.**

The considered documents are:

1. Protocols and design of planned studies.
2. Patient informed consent.
3. Agreement No. 17-75-30035 between the Russian Science Foundation, the principal investigator, and the organisation for the provision of a grant to conduct fundamental and exploratory scientific research.

Twelve people participated in **the vote**. There were 12 votes in favour, none against, and no abstentions.

Resolved: To approve the implementation of the grant RSCF grant No 17-75-30035 "Autoimmune endocrinopathies with multiorgan lesions: genomic, postgenomic and metabolomic markers. Genetic risk prediction, monitoring, early predictors, personalised correction and rehabilitation" at the National Clinical Research Centre for Endocrinology of the Ministry of Health of the Russian Federation.

Committee Chair, Andreeva E.N., doctor of medical sciences, professor */signature/*

Secretary of the Committee, Shatskaya O.A., candidate of medical sciences */signature/*

Локальный этический комитет
ФГБУ «Национальный медицинский исследовательский центр эндокринологии»
Минздрава России

Локальный этический комитет
при ФГБУ «Национальный медицинский исследовательский центр эндокринологии» Минздрава России
117036, Москва, ул. Дм.Ульянова, 11,
тел (499)126-96-70, (495)668-20-79 (доб.4809), e-mail: lek_endocrincentr@mail.ru

---

Выписка из протокола № 17
Заседания локального этического комитета
от «27» сентября 2017 года

Присутствовали:

Председатель ЛЭК:

Андреева Е.Н. – профессор, д.м.н., заведующая отделением эндокринной гинекологии ФГБУ НМИЦ Эндокринологии Минздрава России.

Заместитель председателя ЛЭК:

Шестакова М.В – академик РАН, профессор, д.м.н., директор Института диабета ФГБУ НМИЦ Эндокринологии Минздрава России.

Секретарь ЛЭК:

Шацкая О.А. – к.м.н., ст.н.с. лечебно-диагностического отделения функциональной кардиологии ФГБУ НМИЦ Эндокринологии Минздрава России.

Члены ЛЭК:

1. Арапова С.Д. – к.м.н., в.н.с. отделения нейроэндокринологии и остеопатий ФГБУ НМИЦ Эндокринологии Минздрава России.
2. Беловалова И.М. – к.м.н., помощник директора ФГБУ НМИЦ Эндокринологии Минздрава России.
3. Веденяпина Т.М. – секретарь Российской диабетической ассоциации пациентов.
4. Викулова О.К. – к.м.н., в.н.с., заведующая отделением эпидемиологии и государственного регистра сахарного диабета ФГБУ НМИЦ Эндокринологии Минздрава России.
5. Галстян Г.Р. – профессор, д.м.н., заведующий отделением диабетической стопы ФГБУ НМИЦ Эндокринологии Минздрава России.
6. Зильберман Л.И., к.м.н., ст.н.с. отделения сахарного диабета детей и подростков ФГБУ НМИЦ Эндокринологии Минздрава России.
7. Зиновьева Е.Н. – заместитель директора ФГБУ НМИЦ Эндокринологии Минздрава России по экономическим вопросам.
8. Мельникова О.Г. – к.м.н., в.н.с. отделения терапии диабета с референс-центром обучения ФГБУ НМИЦ Эндокринологии Минздрава России.
9. Шатохина М.С. – главная медицинская сестра ФГБУ НМИЦ Эндокринологии Минздрава России.

Слушали: О проведении экспертизы документации для решения вопроса об одобрении возможности выполнения гранта РНФ №17-75-30035 «Аутоиммунные эндокринопатии с полиорганными поражениями: геномные, постгеномные и метаболомные маркеры. Генетическое прогнозирование рисков, мониторинг, ранние предикторы, персонализированная коррекция и реабилитация» на базе ФГБУ НМИЦ Эндокринологии Минздрава России

Главный исследователь: **проф., д.м.н. Трошина Е.А.**

Рассмотрены следующие документы:

1. Протоколы и дизайн запланированных исследований
2. Информированное согласие пациента
3. Соглашение №17-75-30035 между Российским научным фондом, руководителем проекта и организацией о предоставлении гранта на проведение фундаментальных научных исследований и поисковых научных исследований

В голосовании приняли участие 12 человек «За» проголосовали 12 человек, против и воздержавшихся нет.

Постановили: Одобрить возможность выполнения гранта РНФ №17-75-30035 «Аутоиммунные эндокринопатии с полиорганными поражениями: геномные, постгеномные и метаболомные маркеры. Генетическое прогнозирование рисков, мониторинг, ранние предикторы, персонализированная коррекция и реабилитация» на базе ФГБУ НМИЦ Эндокринологии Минздрава России

Председатель ЛЭК,
профессор, д.м.н. Андреева Е.Н.

Секретарь ЛЭК,
к.м.н. Шацкая О.А.
